# Integrating telecare for chronic disease management in the community: What needs to be done?

**DOI:** 10.1186/1472-6963-11-131

**Published:** 2011-05-27

**Authors:** Carl R May, Tracy L Finch, James Cornford, Catherine Exley, Claire Gately, Sue Kirk, K Neil Jenkings, Janice Osbourne, A Louise Robinson, Anne Rogers, Robert Wilson, Frances S Mair

**Affiliations:** 1 Faculty of Health Sciences, University of Southampton, UK; 2 Institute of Health and Society, Newcastle University, UK; 3 Norwich Business School, University of East Anglia, UK; 4 NIHR School for Primary Care Research, University of Manchester, UK; 5 School of Nursing, Midwifery and Social Work, University of Manchester, UK; 6 School of Geography, Politics and Sociology, Newcastle University, UK; 7 Cranfield School of Management, Cranfield University, Cranfield, Bedfordshire, MK43 0A, UK; 8 Newcastle University Business School, Newcastle University, UK; 9 Institute of Health and WellBeing, University of Glasgow, UK

## Abstract

**Background:**

Telecare could greatly facilitate chronic disease management in the community, but despite government promotion and positive demonstrations its implementation has been limited. This study aimed to identify factors inhibiting the implementation and integration of telecare systems for chronic disease management in the community.

**Methods:**

Large scale comparative study employing qualitative data collection techniques: semi-structured interviews with key informants, task-groups, and workshops; framework analysis of qualitative data informed by Normalization Process Theory. Drawn from telecare services in community and domestic settings in England and Scotland, 221 participants were included, consisting of health professionals and managers; patients and carers; social care professionals and managers; and service suppliers and manufacturers.

**Results:**

Key barriers to telecare integration were uncertainties about coherent and sustainable service and business models; lack of coordination across social and primary care boundaries, lack of financial or other incentives to include telecare within primary care services; a lack of a sense of continuity with previous service provision and self-care work undertaken by patients; and general uncertainty about the adequacy of telecare systems. These problems led to poor integration of policy and practice.

**Conclusion:**

Telecare services may offer a cost effective and safe form of care for some people living with chronic illness. Slow and uneven implementation and integration do not stem from problems of adoption. They result from incomplete understanding of the role of telecare systems and subsequent adaption and embeddedness to context, and uncertainties about the best way to develop, coordinate, and sustain services that assist with chronic disease management. Interventions are therefore needed that (i) reduce uncertainty about the ownership of implementation processes and that lock together health and social care agencies; and (ii) ensure user centred rather than biomedical/service-centred models of care.

## Background

Since the beginning of the 1990s, telecare systems - information and communications technologies that link people (usually at home) to health and social care services - have been promoted as a technological solution for problems of equity and access to care, and as a means of support for self-care in the community. Telecare systems are attractive to health and welfare agencies because they allow people with long-term illnesses to be remotely monitored, or to monitor themselves, at home. Such systems have been aimed at providing responsive services for people with chronic illnesses such as chronic obstructive pulmonary disease (COPD), diabetes, and heart failure. Systematic reviews show that telecare systems can be used effectively to do this work [1-4]. An important objective of such systems has been remote monitoring of symptoms to provide an early warning of exacerbation events or deterioration, and to prevent hospital admissions. In the same period, generic systems aimed at ensuring the safety and security of frail older people have also been intensively promoted as a response to the anticipated increasing demands of such people on health and social care services, and as a means of controlling the costs of such services. These systems have come to be incorporated in policy in the UK as a means of combining self-care, symptoms surveillance, and social support [5-7]. There is evidence that telecare systems can be used effectively to support frail older people in their homes (telecare), to prevent or delay admission to residential care, and to monitor conditions with the aim of secondary prevention[8,9]. However, this evidence is not unequivocal, and its interpretation by practitioners and policy-makers is shaped by multiple political and organization factors [10]. Furthermore, there remains much to understand about how such systems reconfigure existing practices and relationships [11], and how best to translate trial results into routine practice also remains unclear [12].

Previous research in this area has been dominated by small scale case studies and medical perspectives. Robust literature reviews have added to our knowledge about the effective organization and delivery of telemedicine services in specialist clinical settings [13-15]. An important result of this literature is that we know a good deal about the role of specific factors in the implementation of relatively small-scale tele*medicine *services, but much less about the implementation of large scale multi-agency tele*care *services. The aim of this study was therefore not to return to the specialist clinical services that we had previously investigated in detail, but instead to explore telecare as a tool for chronic illness management at the intersection of health and social care services and with patients. Although home telecare systems appear to offer promising solutions for services that are faced by constraints on resources, they also seem to have suffered similar problems of integration and workability to those experienced by specialised clinical telemedicine systems [16-18]. The implementation of telecare has been slow and uneven, even though it has been actively promoted by government for more than a decade, and despite large scale demonstration projects with positive outcomes [15]. Our aim in this study was therefore to identify the policy and practice factors that affect the routine incorporation of telecare into everyday practice, and to explore the ways that these factors promoted or inhibited the implementation and integration of telecare systems. Further, we wished to use qualitative methods to investigate a wide field of policy and practice from a 'whole systems' [19] perspective, and to understand why multiple attempts to implement telecare have not led to it becoming an integral part of the management of chronic disease in the community.

The study reported in this paper had two objectives. First, we sought to identify, describe and understand those factors that promote or inhibit the implementation and integration of telecare systems for chronic disease management in the community, with reference to the views of four key stakeholder groups: patients and carers; healthcare managers and professionals; social care managers and professionals; and telecare systems manufacturers and suppliers. Second, we sought to identify a set of principles, grounded in the experiences and perspectives of participants, which could be used to inform policy and practice around telecare implementation in the context of a 'whole systems' approach [20]--that is, across boundaries of the private, public and domestic sectors, all of which are playing an increasingly important role in the management of chronic disease [21]. The study reported here may be the largest and most comprehensive qualitative study in this sphere to date.

## Methods

The aim of this study was to understand the general dynamics of service implementation and integration across a range of settings, and develop from the ground up principles to inform policy interventions [22]. The study was organized along federal lines, with work-packages associated with the perspectives of different sets of participants, each of which was associated with the interests of groups of researchers within the study. After receiving Ethics Committee approval the study was undertaken between 2007 and 2009 across England and Scotland. Approval was obtained from Newcastle and North Tyneside 2 Ethics Committee (Ref 07/Q0906/52, 29 May 2007).

### Sampling and Recruitment

To provide a foundation for the study, we undertook key informant interviews with participants who offered a strategic view of problems related to telecare implementation and policy context. Using contacts made in earlier studies, we recruited a sample of managers in primary care (n = 9) and social care (n = 13), who played a policy role in commissioning, organizing and delivering telecare services, and representatives of the service supply and manufacturing sector (n = 11) who sought to promote telecare systems to health and social care providers.

Although we did not intend to investigate or evaluate specific services we sought to recruit participants who had operational experience of telecare implementation and integration processes. To ensure appropriately experienced health and social care professionals, and patients, 'on the ground', we identified a maximum variation sample of nine telecare services in England (n = 7) and Scotland (n = 2) from which to recruit them. Variation was according to:

(i) **service provider**: National Health Service (n = 4), Social Care (n = 4), or Inter-agency collaboration (n = 1);

(ii) **service type**: self-monitoring of symptoms for effective self-management and reduction of demands on primary care for Coronary Heart Disease, Chronic Obstructive Pulmonary Disease (COPD), and Diabetes (n = 4) vs remote monitoring at home of older people with multiple comorbidities and cognitive impairment to prevent admission or readmission (n = 5); and

(iii) **service history**: planned services (n = 1), experimental or demonstration projects (n = 3), or established services (n = 5).

To recruit to task group and workshops we sampled primary care professionals (n = 30) and social care professionals (n = 60) associated with each of these sites. Manufacturers and suppliers of telecare systems are crucial to their successful implementation and integration. We were able to identify participants who were associated with three of our research sites. Because we were not evaluating these sites but rather using them as vehicles to identify knowledgeable participants in the study we then sampled outwards from these participants using their social networks and recommendations to obtain a wider range of experiences of different services. This chain referral [23], or 'snowball' sampling strategy led to the recruitment of 67 participants from this previously under-researched group. Only 4 participants did not have direct experience of telecare services, and these were associated with a service that had only reached the planning stage. Many had experience of large scale services, one involving in excess of 2000 users; and others had experience of wide-ranging telecare services from different telecare providers across a single geographical region.

We purposively recruited 31 patients and carers into the study. Of these five (associated with a web based tool used for the management of COPD) took part in a focus group and 26 took part in individual interviews. Of the 26 interviewed, 7 were carers (2 of whom were interviewed in place of their relative who felt too ill to take part; and 5 participated jointly with the patient). Most of our participants were older people with multiple chronic comorbidities and all interviewees were in extremely poor health, hence making individual interview the most appropriate method. A further factor complicated recruitment of service users. In earlier studies we had noted that this group were frequently excluded from research on operational aspects of telecare. This situation has changed, and it meant that frail older people from four sites included in this study were already involved in research or evaluation studies being undertaken by other universities. They were thus excluded from the study reported here. This means that our sample is composed of people who were using symptom surveillance and management systems (Asthma, Coronary Heart Disease, COPD, and Diabetes) in the community. Other groups have reported findings that relate to cognitively impaired users of telecare services in very similar settings [24-26].

### Data collection

Individual semi-structured interviews were undertaken with key informant health and social professionals, patients and service suppliers and manufacturers. In each case, participants were approached by email or phone and invited to participate, were provided with a study information sheet and gave informed consent prior to interview. Individual semi-structured interviews were also undertaken with 26/31 service users. They were approached by letter sent from their family practitioner on behalf of the research team, and returned a signed informed consent document before being approached by a field researcher. All interviews were conducted in the participant's office or in the patient's home. Interviews lasted between 45 minutes and two hours. An interview schedule was used to guide interviews, which were audio-taped and fully transcribed.

Because an aim of the study was to develop a set of principles to inform policy, we used task groups [27] as an opportunity for participants to work together to discuss and create these. In these groups, participants were encouraged to be creative, and to think beyond the confines of their professional orientation to service implementation and integration problems. It proved difficult to attract representatives of service suppliers and manufacturers to small focus groups, but in collaboration with two trade associations we held two well attended workshops. In both of these workshops large groups of participants broke down into smaller groups (ie. task groups) and worked together to build sets of principles. Task group size ranged between three and eight members. We collected qualitative data by audio-recording and observing group discussions, but also photographed their work in progress when this involved flip charts, drawings, or service maps.

### Data analysis

Qualitative data collected in interviews and task-groups consisted of verbatim transcripts, and detailed field-notes. Transcripts were first checked against original recordings for accuracy, and initial analysis was undertaken by field researchers associated with each of the work-packages of the study, using conventional thematic coding techniques [28] that identified and described the perspectives and experiences of participants. Data analysed in this way was then summarised and presented in reports to the whole research team, which met regularly in 'data clinics' to interpret analytic outcomes. Data clinics were themselves audio-recorded and transcribed to ensure that group analyses were preserved.

There was a further body of data, and this consisted of the principles for action that were generated by group and workshop participants. Eighty-six principles were generated during task groups and patient interviews. These were edited and reduced to 75 after the elimination of duplicates. Both kinds of data were then subjected to an integrative analysis, and informed by Normalization Process Theory (NPT) [29,30]. Here data (which now also included transcripts of data clinics, and sets of policy principles) was re-coded within a theoretical framework [31] that reflected key constructs of NPT. This revealed to us specific factors that promoted and inhibited the implementation and integration of telecare systems for chronic disease management in the community, and related these to underlying mechanisms at work.

**Figure 1:**
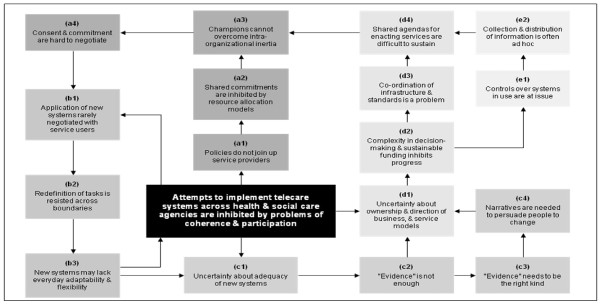
**Uncertainty is continuously cycled through telecare domains**.

Once this qualitative integrative analysis was completed we undertook a modelling procedure [32] in which we presented these factors as 'nodes' in a network of events that could be mapped in relation to each other (see Figure 1). Its aim was to characterise organizational processes at work in terms of contingencies and their consequences and this should not be viewed as a sequential flow chart as the order of events may vary, some occurring in parallel so this is not meant to represent a step by step guide, nor are different weightings provided to different aspects.

## Results

If telecare is the answer to supporting care for people with chronic illness in the community, then what needs to be done to properly integrate it with existing organisational and professional mechanisms for doing this work? Our study revealed the ways that multiple cycles of uncertainty run through implementation processes and inhibit the embedding and integration of new ways of delivering care.

In Figure 1 we present a model of the analysed data that shows how uncertainties were derived from problems of coherence and participation. This follows the key storylines that ran through participants accounts. Along each storyline are a series of nodes that identifies a factor that inhibits the normalization of telecare systems in practice and which appeared in respondents accounts. The model thus defines the cumulative relationships between factors that serve to inhibit embedding of new technologies and their associated ways of working in practice, and so reduce the scope for integration in everyday service delivery. In what follows, we work through these storylines.

### Storylines *a*→*b: *Policies do not join up local service providers

We found evidence of problems of engagement across the boundaries of health and social care agencies, and both service suppliers and social care professionals emphasised that primary care professionals and managers were often indifferent and sometimes hostile to the implementation of telecare systems. Across our stakeholder groups, there were mixed perspectives concerning the role of policy and its facilitative (or prohibitive) effect on achieving integration of telecare across boundaries (node a1). From the perspective of the Supplier group, the perceived role of the government generally, and of the UK Department of Health in particular was in dispute. For some, the *'Government have done lots to develop a telecare market'*, but this was contradicted by others: '*The government has not done much.' *More generally there was concern that government support for telecare was not coherent and well joined up and that there was a lack of leadership on this within government. One suggestion from the Supplier group was that there should be a 'Telecare Tsar', someone charged with insisting that telecare be taken up and rolled out.

From the Health Professionals, a common view reflected this more prescriptive role for policy, suggesting that policy is only useful if it makes telecare *mandatory:*

*"I think, unfortunately, there has to be some framework or legislative process that forces health boards and the managers of trusts to actually take this forward and take it seriously, put money into it"*.

However, some health professionals felt that if the service was clearly effective, then it would be taken up regardless. For some, the translation of policy into official telecare implementation guidelines would facilitate the movement of telecare into mainstream healthcare (node a1):

"....We certainly need guidance and support around the IT infrastructure and the IT issues that we may face and the challenges that are around that as well. There's a whole list of things that we could go through but I think some kind of national strategy on telecare and a national structure and national support and guidance. How to do lists, you know, a full guide to telecare"

On questioning about the role of telecare policy in relation to specific health care initiatives - such as (British) National Service Frameworks and Quality Outcome Frameworks - some health professionals felt that clearly connecting supportive telecare policy with service initiatives would facilitate integration, but others expressed concerns that lack of 'joined up' working across different sectors of the NHS could introduce problems (node a2), for example:

"I think it [integrating telecare across service divisions] could be seen to support it [chronic disease management] but I also think it could have an adverse effect on it and I think one of those things we don't do quite so well is communicate with GPs what targets have been agreed by the patient. So, you know, you might have an elderly gentleman of 85 living on his own. Well, you really don't want an HbA1C less than 7.4% if he's on life doses of insulin because he's probably not very safe then. So I think we need to communicate back perhaps just to say we've agreed a target HbA1C of whatever, because otherwise their reading my bloods and thinking,...in that service that group of commissions we're paying for, what are they doing?"

Problems of an absence of policy directives, or unhelpful specification of such directives, in relation to telecare led to difficulties in maintaining shared commitments across sectors, due to incompatible resource allocation models (node a2). From the Suppliers group, participants noted that many of the companies currently active in the development of telecare and telehealth equipment are innovative SMEs, and they face typical cash flow problems:

"...the majority of the companies involved are SMEs with precious little cash flow, very little reserves and I see their managing directors, chief execs on a regular basis and some of them say to me when they get up in the morning they never know whether by the time they go to bed at night they've still got a business."

For some Supplier participants, these problems were compounded by the focus on the National Programme for IT in the health service, which at the time of this study had taken the focus away from technologies such as telecare and away from SME suppliers in favour of large system integrators: *'NPfIT were a problem because they destroyed the market for SMEs'*. For many of these equipment manufacturers and designers there were also questions about how to market their products and who to market them to. We turn to these next.

### Storylines *d*→*b: *Ownership and direction of business and service models is uncertain

Participants described the ways that uncertainties about leadership, ownership, and responsibility disrupt the field of telecare. They pointed to the ways that uncertainty about leadership retards co-ordinated policies to develop services, and that uncertainty about ownership and responsibilities means that appropriate business and service models are hard to define.

From the perspective of Suppliers, a central issue is the lack of integration of health and social care (node d1), often generating separate markets with different rules and regulations, different bodies of professional knowledge, different criteria of evaluation, different technical expectations, different funding models and different cultures. It was often not clear who the institutional customer for Telecare was - health or social care: the one who paid the costs or the one who received the benefit? Another issue here was the lack of professionals with both health and social care knowledge, but this was also seen as confusing for the patient/customer:

"One of the problems we've got as well is GPs can prescribe assistive technology, Home Improvements can prescribe and the voluntary sector can prescribe and DH can prescribe them through grants and local authorities and that means for the end consumer knowing where to go - actually where do you go? ... In some areas the GP will direct you to a service, in other areas you've got the voluntary sector [...]and there is a bit of a mess in terms of knowing the end user, what you have to pay for, what you can be delivered, [and] what can be privately [provided]"

A key problem resulting from uncertainties about ownership and responsibility concerned a frequent lack of sustainable funding, which inhibited progress (node d2). Health professionals in particular highlighted this concern, as most of the telecare services were started as a direct result of specific funding initiatives. Funding was seen as necessary to address issues such as purchasing equipment, recruiting additional staff and conducting evaluation studies. However while it was viewed as a necessity it was not viewed as the most important factor.

"There is a huge need for obviously the funding to develop the pilot and buy new equipment. Also there's a huge need for funding around the evaluation and the research into this project because we're all having to go and find this money from somewhere"

Issues concerning funding - and in particular, incompatible commissioning processes across sectors - were expressed by Supplier participants (nodes d3, d4 - a3), for whom working with the various parts of the NHS presented another set of problems. The need to involve primary care, and in particular GPs, in telecare was widely expressed. Some felt that changes in commissioning would bring in GPs. As one interviewee put it: *'GPs are a problem but practice based commissioning should change that'*. Many of the Supplier participants reported problems engaging with Primary Care Trusts which, at the time, were responsible for commissioning services. For example: *'There is a problem with PCTs not being geared up'*, and *'...need to get the commissioners in PCTs onboard as nothing will happen without them'*.

Health professionals also referred to problems of engagement, and the difficulty of establishing and maintaining shared agendas across these boundaries, but placed some emphasis on the development and deployment of technical systems appropriate to service requirements (nodes d2-e1 & e2 - d4). While some had a good relationship with their suppliers, many professionals felt that a lack of engagement with suppliers was a barrier to telecare implementation. It was felt that there needed to be some dialogue between health professionals, suppliers and manufacturers so that the technology provided met professionals' requirements.

"One of the problems was company X [...] we had all these promises of a bespoke system but what they gave us was something that they had already and they tarted it up a bit, so they gave us this call/contact centre, which is a call centre system, and they tweaked it, didn't they?"

The kind of problem referred to above reflected more general uncertainty expressed amongst health professionals about the supplier market, as some participants felt that the market for products was small and that different companies often sold the same thing. Additionally there were concerns that some suppliers were also funding the research and were often good salesmen and thus there was a lack of unbiased advice. Health professionals were often unsure of the range of technologies and what should influence their choice of technology, and of the right supplier to provide it:

"We need an idea of the companies that are out there. We need support and guidance around accessing these companies and getting the information"

Health professionals themselves indicated that the problem of attaining shared agendas for enacting services (node-d4) was as apparent within their sector as beyond it. There was a general consensus that team working was necessary for successful integration and implementation of telecare, both within internal teams and between internal and external teams:

"I guess you'd require appropriate backup and require the rest of the multi-disciplinary team to know that that's the nature of the consultation. Sometimes I think you would have limited information from the telecare service compared to if it was a face to face consultation. You know, I think that has to be known across the whole of the contacts that that patient has thereafter".

In some cases a lack of dialogue between primary and secondary care teams presented difficulties in shifting the balance from secondary to primary care thus hindering telecare progress. Additionally it was felt that there needed to be improved communication between health professionals and IT staff who often did not understand what each was trying to do.

"Yes, I think what there has to be is a real dialogue between the people within IT, not necessarily the IT support, but the e-health staff and the clinicians, and I think that's one of the problems"

System manufacturers and suppliers were therefore anxious about the structure of the market itself, and emphasised that health and social care services often lacked clear business and service models to sustain telecare in practice.

### Storylines *b*→*c *New systems are rarely negotiated with service users

Participants from all stakeholder groups (except service users themselves) emphasised the need to connect service-centred policy and user-centred practice. Supplier participants acknowledged a general lack of focus on the end users of telecare, and indicated during workshop activities, that new models that were 'user centred' were required. This general lack of understanding of the diversity of needs of telecare users was noted by one supplier:

"...[that's an] important point there actually we assume there is a lot of diversity in the younger population, but it's often assumed that you get less diverse as you get older but it's the opposite actually you get much more diverse..."

Similar arguments were made by Health Professionals, who emphasised a frequent 'mis-match' between telecare systems and their service configuration and the characteristics of individual patients (nodes a4 and b1) (referred to by some participants as issues of 'non-compliance'), suggesting for example, that:

"Some patients are quite comfortable and happy with technology and other patients aren't. They much prefer the human interaction. Some patients are comfortable with the responsibility and autonomy and other patients aren't."

In suggesting solutions to problems of integrating telecare, Health Professionals emphasised the importance of matching individual patients with use of particular telecare systems, highlighting the need for flexibility and choice (node b-3), because of it not being suitable for some 'types' of patients. Such concerns however, extended beyond 'case by case' assessments to reflect a more general problem of not adequately tailoring telecare systems to the local context. As one Health Professional commented:

"I mean it's been quite difficult ... obviously, it's been set up although based on a different service. To then mirror that into sort of a primary care service within a different city is a very different sort of ... it's essentially going to be based on the same sort of service down south, to then sort of try and apply that to a different city, and the way that different city's healthcare works within that city, has been a wee bit difficult"

It was felt that integration and implementation would be smoother if professionals had the opportunity to select and design services specifically geared to the local context and aims. Additionally, some suggested that patients, as experts on their condition, should be able to pick and choose technology according to their needs. Although we encountered many different ways of thinking through the implications of telecare systems in practice, and expressions of support for the need to more adequately address the needs of service users, there was little evidence of attempts to consult and include patients and carers in these processes (node b1-b3). This was reflected in comments made by service users themselves, many of whom reported not being forewarned as to *how *the telecare could impact on the home environment and in particular interference with the operation of existing home technologies (e.g. TV, flashing lights). Equally, most patients found ways of dealing with this themselves, which involved experiential learning (i.e. trial and error approach) about how to use the device, for example:

"No, odd times it blips...I call it blipping. Like when I put my finger in the probe for the blood oxygen, oxygen in the blood er, it will shoot up to ninety nine, it's never been ninety nine, now it, obviously, I can tell that that's not going to work that, so what I do is take my finger out and do some of the others and I go back to that and try again so you know..."

Such 'glitches' with equipment were usually managed. However the interviews with service users suggested that there was generally little scope for the user to individualise the system in a way which best suited their individual needs or the ways in which they had previously learnt to manage their condition. Rather the workings of the equipment forced the user into adapting to the workings of the machine.

Importantly, this lack of negotiation with service users meant that professionals in health and social care, and service suppliers often underestimated the degree to which patients and carers were already involved in self-care, and the burden of work that followed from it. Thus, in some ways, the level of re-definition of tasks across boundaries (node b-2) was not always as great as assumed by professionals. Prior to taking part in the telecare service most of the participants (there were a small number of exceptions) managed their condition following a traditional biomedical approach comprising of medication and self-surveillance. Thus, telecare meant a 'stepping up' of what they were already doing and for most people the telecare system provided reassurance rather than making any significant change or integrating it in a way that extended patient initiated care, as reflected in a comment from one service user:

"...Basically, I mean, what this system has done is emphasised and built on the previous knowledge I had, um, and has made me much more aware of my condition daily...And so it confirms okay, that I'm feeling better or I'm not feeling better having a good day or a bad day, um, but it gives you that feeling of security to know that somebody else is also looking..."

Although not necessarily re-defining the tasks required of home users, there was some uncertainty about 'the point' - in health benefit terms - of collecting the kind of information that was demanded by these telecare systems:

"It is so basic, it's stuff that actually is already known to your practice, they know if you smoke, they know what you do, you know it is fairly pointless .... It struck me that it wasn't actually a great deal of use um, because you need a peak flow reading and a comparison for that peak flow."

For most service users we interviewed, this apparent lack of sense of purpose - although reflecting again a lack of negotiation with service users - did not matter too much to them. What mattered was that they were engaging in what was being asked of them by the health care providers, and the trade-off, from their perspective, was that they had a legitimate (and faster) route to access to professional care as and when required.

### Storylines *c*→*e: *Uncertainty about the adequacy of new systems undermines user confidence

Participants in this study saw an urgent need for evidence that would convince senior decision-makers that telecare was a viable alternative to existing patterns of 'in-person' service delivery. From the perspective of Health Professionals, lack of national evidence was a major barrier put forward by members of all the professional groups as to why telecare had not become part of mainstream healthcare (node c-2 & c-3). As expressed by one of our participants:

"there has to be some kind of evidence that intervening this way makes a difference. You know, that that's quite important as well. So there's no point in monitoring people if there's no evidence that monitoring actually prevents admissions, so obviously I think it's probably quite important that you invest in these things which are likely to work,"

There were others who expressed views that evidence of telecare benefits was not enough to change professionals' opinions and that such evidence would have to be significant.

"It has to deliver. I mean, sometimes people get seduced by technology and by promises of what might be and so often with technology and IT and so on it's a disappointment, it's an anticlimax. So I think the most important thing for telecare is that it has to deliver real benefits. It's not just for it to be a different way of doing something, almost for the sake of it, and I think it should add some value to the system which should be safer, or cheaper or easier, or some other advantage, not just it's technology"

For suppliers too, 'evidence' was seen to have a key role, but that different kinds of evidence were important (nodes c1-c4). They placed much emphasis on 'success stories' to promote telecare and persuade people that it is worth investing time and effort into making telecare 'work'. For all professional stakeholder groups, 'champions' were still seen as the key agents of persuasion in making the case for telecare. This perspective is summed up in the following quote from a Supplier:

"I think it's, this funding thing is a big problem. I think some creativity amongst groups of people who are enthusiastic about telecare is quashed, people are asked to do risk analysis, people are asked to do an analysis on what the benefits will be they're almost having to commit themselves to deriving a benefit for their particular department should they buy this stuff. If they don't buy this stuff then they're not putting themselves in a situation where they could be chastised for getting it wrong that's a problem so creativity is stifled, if you like, by people who want to live within their comfort zone. You also see evidence of people really getting excited about it on their own but know they'll have difficulty selling it to somebody else, know how much time it's going to take and draw back from their enthusiasm because it's too time consuming for them to actually take it forward."

Suppliers and Social Care Professionals were less impressed with evidence derived from clinical trials - the large scale 'academic' studies that health professionals argued were necessary to demonstrate the safety and effectiveness of telecare services. Here, social care professionals and service suppliers sought mechanisms to systematically collect data about the performance of services in practice that would be meaningful in making the case for telecare *within *their organizations, rather than at a policy level. They aimed to enable robust claims about the comparative utility and cost effectiveness of telecare systems as mainstream, not experimental, services and enable managerial comparisons with other forms of service provision. Until that was possible, for many participants, the jury remained out on telecare.

## Discussion and Conclusion

This study has highlighted key obstacles to the implementation and integration of telecare systems for chronic disease management, within existing patterns of community based health and social care delivery. Through use of a "whole systems" approach we have shown that at the level of service design and delivery, organizational links between policy and practice lack coherence. Service manufacturers, suppliers, and providers all struggled with uncertainties about who, in practice, was responsible for implementing telecare, and patients and service users made sense of telecare in ways that differed from the assumptions made by their health care providers.

In this study, multiple stakeholder groups described problems of ambiguous or incompatible policy directives in relation to telecare service provision. This suggests that strengthening links between policy and practice may facilitate the integration of telecare and increase participation--perhaps through changing the structure of GP remuneration and the key targets of the Quality Outcomes Framework. Within their own organizations, participants emphasised the important role of champions in securing readiness and organising change management, a finding consistent with much of the existing literature, but for which assumptions have recently been challenged [33]. However, this focuses attention on individual leadership rather than the ways in which intra-organizational inertia can be structurally induced. Our data as a whole suggests that whilst 'champions' can be important facilitators, maintenance of structures to support the ongoing provision of telecare requires much more. The lack of shared organisational vision that stems from the absence of coherent policy encourages different groups of professionals to see each other as barriers to, not facilitators of, change.

A whole systems approach to telecare for chronic disease management will also require require addressing uncertainty about ownership and direction of business and service models. In our study, telecare manufacturers and suppliers saw the absence of sustainable service models as a threat to their industry, and to their capacity to deliver systems to the public sector. Such uncertainties make it difficult to operationalise services in practice. This is not necessarily a matter for policy leadership, but rather for agreements about local responsibilities. The question of direction needs to be answered, and mechanisms for joining together health and social care agencies as telecare service providers must be developed.

Lack of negotiation with service users in configuring new systems remains problematic, and presents a barrier to more widespread uptake and integration of telecare services. Devising and introducing new systems needs to take account of how individuals currently manage conditions and the ways that they adapt to their chronic illness. Understanding the fit between the everyday routines of service users and technologies in the home is essential if uptake and use of telecare is to develop. While professionals and service suppliers sought policy direction and resources - focusing on biomedical or service-centred models of telecare, patients and carers were already using these systems in unexpected ways. They did not necessarily appreciate them as self-care technologies, but instead valued them highly as ways of demonstrating their co-operation with health care providers and as means by which requests for personal healthcare could be demonstrated to be legitimate and warrantable once they had passed the thresholds objectively set by the telecare system itself. More resources may not be the answer to mainstreaming telecare services, but understanding the ways that they change the work and workload of being a patient, primary care professional, or social care professional, do have an important part to play. Mainstreamed services may actually *increase *demand and make it difficult for health professionals to negotiate calls for care, because clinical information obtained through self-monitoring can be used to legitimate calls for attention from health and social care professionals.

This study advances on previous work in three ways. First, it offers a broad and comparative analysis of different services and settings, in a field where - as a recent Cochrane review [34] points out - robust evidence is lacking, both about the conditions necessary for effective implementation, and about the operationalisation of new technologies in broader multi-disciplinary settings. Here, we have not simply examined integration and implementation issues within particular service provision contexts, but rather have explored and identified key barriers and facilitators to realizing telecare as a 'whole system' in which the domestic and other contexts are differently experienced and configured as contributing to chronic disease management. This is of increasing importance, as maximising the effectiveness of technology for disease management beyond formal settings demands greater understanding of the significance of shifting locations of care, and associated shifts in power relations that technology-facilitated disease management may present [35]. Second, by focusing on the wider organizational experience of participants as they seek to operationalise new ways of providing care we have shifted attention away from a medical model of service provision. Much of the existing literature around interdisciplinary boundaries and information technologies has focused on informatics rather than telecare, or has investigated interactions between specific professional groups (often doctors and nurses) rather than exploring 'whole systems' at work [36].

Finally, this study breaks new ground as it highlights barriers and facilitators to telecare integration through research involving the full range of stakeholders/actors, including service manufacturers and suppliers, health and social care professionals and managers as well as end users. Thus this work provides insights beyond those of studies which have, for example, sought an overall assessment of telemedicine or telecare services from a single key individual [37] or systematic reviews of primary studies, which have not taken this whole systems perspective [13]. It has highlighted issues such as problems of uncertainty about ownership and direction of business, and service models as a key problem not addressed in the previous literature [38,39]. It adds further weight to the argument that 'proof of concept' trials have not added greatly to the evidence base to support telemedicine and telecare--we have argued for some time that they are unlikely to do this [40].

Nonetheless, this study has a number of limitations. We did not investigate in depth the operation of specific services, but rather used them as vehicles to identify a heterogeneous sample of participants. This overcame an important problem - some earlier studies have found it difficult to recruit participants who have actually been involved in providing telemedicine and telecare services in practice [41]. Funding and logistics meant that we could not undertake longitudinal ethnographic work, or examine outcomes for service users. Our interviews with the latter focused on people using services aimed at monitoring and managing specific symptoms and, once again, for logistic reasons, we did not explore the experiences of older people using home safety services or movement sensors. However, although this is a single phase study it covers important ground for the first time. In moving beyond previous work that has been dominated by a medical model, this study takes an important step forward by including - for the first time - the perspectives and experiences of patients and carers, social care managers and professionals, and manufacturers of suppliers. It shows how those supplying, organizing and delivering telecare systems in practice struggle with multiple cycles of uncertainty - even in well-established and apparently well integrated services.

This work has clear implications as it suggests that interventions are needed that reduce uncertainty about the ownership of implementation processes and promote development of a shared vision. These were reflected in uncertainties about how different professional communities of practice could be best incorporated into the development of telecare services for larger populations. There was little evidence in our study of shared commitments to the development of telecare services across the boundaries between health and social care and of shared understandings of the potential role of telecare between different user groups. There was, however, evidence of some antagonism and tensions across these boundaries. Thus, interventions are needed that establish communities of practice bound by shared ideas about common cause and thus to improve the outcomes of implementation processes. Furthermore, there is a need to move from biomedical and service centred models of care to user centred models of care which acknowledge that the implementation of telecare systems owes as much to the work of patients as it does to formal health and social care agencies.

## Authors' contributions

The study was conceived and designed by CRM, TLF, JC, SK, LR, AR, RW and FSM. Data was collected by all authors and analyses were conducted by CRM, TLF, JC, CE, CG, SK, KNJ, JO, LR, AR, RW and FSM. This paper was drafted by CRM, FSM, and TLF. The guarantor of this paper is CRM. All authors read and approved the final manuscript.

## Pre-publication history

The pre-publication history for this paper can be accessed here:

http://www.biomedcentral.com/1472-6963/11/131/prepub
